# Advanced glycation end products inhibit the osteogenic differentiation potential of adipose‐derived stem cells by modulating Wnt/β‐catenin signalling pathway via DNA methylation

**DOI:** 10.1111/cpr.12834

**Published:** 2020-05-28

**Authors:** Yong Li, Lang Wang, Maorui zhang, Kui Huang, Zhihao Yao, Pengcheng Rao, Xiaoxiao Cai, Jingang Xiao

**Affiliations:** ^1^ Department of Oral and Maxillofacial Surgery The Affiliated Stomatology Hospital of Southwest Medical University Luzhou China; ^2^ State Key Laboratory of Oral Diseases West China Hospital of Stomatology Sichuan University Chengdu China; ^3^ Orofacial Reconstruction and Regeneration Laboratory The Affiliated Stomatology Hospital of Southwest Medical University Luzhou China; ^4^ Department of Oral and Maxillofacial Surgery The Affiliated Hospital of Southwest Medical University Luzhou China; ^5^ Department of Oral Implantology The Affiliated Stomatology Hospital of Southwest Medical University Luzhou China

**Keywords:** adipose‐derived stem cells, advanced Glycation End Products, DNA Methylation, osteogenic Differentiation Potential, Wnt Signalling Pathway

## Abstract

**Objectives:**

Advanced glycation end products (AGEs) are considered a cause of diabetic osteoporosis. Although adipose‐derived stem cells (ASCs) are widely used in the research of bone regeneration, the mechanisms of the osteogenic differentiation of ASCs from diabetic osteoporosis model remain unclear. This work aimed to investigate the influence and the molecular mechanisms of AGEs on the osteogenic potential of ASCs.

**Materials and methods:**

Enzyme‐linked immunosorbent assay was used to measure the change of AGEs in diabetic osteoporotic and control C57BL/6 mice. ASCs were obtained from the inguinal fat of C57BL/6 mice. AGEs, 5‐aza2′‐deoxycytidine (5‐aza‐dC) and DKK‐1 were used to treat ASCs. Real‐time cell analysis and cell counting kit‐8 were used to monitor the proliferation of ASCs within and without AGEs. Real‐time PCR, Western blot and Immunofluorescence were used to analyse the genes and proteins expression of osteogenic factors, DNA methylation factors and Wnt/β‐catenin signalling pathway among the different groups.

**Results:**

The AGEs and DNA methylation were increased in the adipose and bone tissue of the diabetic osteoporosis group. Untreated ASCs had higher cell proliferation activity than AGEs‐treatment group. The expression levels of osteogenic genes, *Opn* and *Runx2*, were lower, and mineralized nodules were less in AGEs‐treatment group. Meanwhile, DNA methylation was increased, and the Wnt signalling pathway markers, including β‐Catenin, Lef1 and P‐GSK‐3β, were inhibited. After treatment with 5‐aza‐dC, the osteogenic differentiation capacity of ASCs in the AGEs environment was restored and the Wnt signalling pathway was activated during this process.

**Conclusions:**

Advanced glycation end products inhibit the osteogenic differentiation ability of ASCs by activating DNA methylation and inhibiting Wnt/β‐catenin pathway in vitro. Therefore, DNA methylation may be promising targets for the bone regeneration of ASCs with diabetic osteoporosis.

## 1 INTRODUCTION

Diabetes and osteoporosis are common diseases in aged populations, and numerous studies have demonstrated a close relationship between diabetes and osteoporosis.^1^, ^2^, ^3^ Diabetic osteoporosis is one of the most important complications of diabetes mellitus in the skeletal system and is a systemic metabolic bone disease accompanied by osteopenia and destruction of bone microstructure leading to a high risk of fractures. The incidence of diabetic osteoporosis occurs in about 50% of diabetic patients, and the risk of osteoporotic fractures is significantly increased in patients with diabetes compared with healthy subjects.^1^ Recent studies showed that the increased incidence of fractures in diabetic patients is associated with the long pathogenic effects of diabetes. Now, many researchers focus on the issues of reducing bone damage and promoting bone regeneration in diabetic patients.

Promising bioengineering technologies, such as tissue engineering, which can repair bone defects with stem cells, may provide novel tools for reconstructive surgery. Recent studies reported that adipose‐derived stem cells (ASCs) have been widely used in bone tissue engineering and regenerative medicine research.^4^, ^5^, ^6^, ^7^, ^8^, ^9^ ASCs are ideal stem cells because they can be harvested easily from adipose tissue. Moreover, they have the capacity to differentiate into osteoblastic‐like cells, extensive proliferative ability and low immunogenicity.^10^, ^11^, ^12^ Diabetes mellitus can seriously affect the metabolism of bone tissue. Roy, B’s research showed that diabetes leads to the dysfunction of osteoblasts and osteoclasts, and have negative effects on the differentiation of stem cells into bone cells.^13^ The osteogenic potential of ASCs might be destroyed in the high glucose and inflammatory environment caused by diabetes.

Advanced glycation end products (AGEs) are the end products of glucose, fructose, glucose 6‐phosphate and proteins via a non‐enzymatic and non‐reversible glycosylation reaction. The formation and deposition of AGEs accelerate during ageing, inflammation and especially diabetes.^1^, ^14^, ^15^, ^16^, ^17^, ^18^ Schwartz et al and Ding et al^16^, ^17^ found that AGEs significantly inhibit the proliferation of osteoblasts but increased the activity of osteoclast, which eventually leads to reduced bone strength, osteoporosis and fractures. Therefore, AGEs are associated with the development and progression of diabetic osteoporosis. However, there is no clarity concerning the mechanisms whereby AGEs lead to diabetic osteoporosis.^1^, ^14^, ^15^, ^16^, ^17^, ^18^

DNA methylation refers to the methylation of CpG dinucleotides on cytosine dinucleotide 5‐carbon atoms under the action of DNA methyltransferase using S‐methionine as the donor of the methyl group. Recent studies show that hypermethylation of a DNA sequence is associated with the suppression of gene expression and demethylation of DNA is contrary to the effect of DNA methylation, which leads to an increase in gene expression.^19^, ^20^, ^21^, ^22^ DNA methylation modification affects the normal differentiation function of cells and has a close relationship with the occurrence, development and treatment strategies of diseases. Studies showed that DNA methylation modification during stem cell differentiation is apparent in a variety of bone diseases, including osteoporosis and osteoarthritis.^23^, ^24^, ^25^ In the process of osteogenic differentiation of stem cells, DNA methylation is a mechanism involved in regulating the expression of osteogenic marker genes, and the expression of stem cell differentiation‐related genes is inhibited because of the high promoter methylation levels. However, there are only a few reports describing how AGEs regulate DNA methylation in ASCs.

Based on literatures and our previous work, we put forward that AGEs might have negative impact on the physiological functions of ASCs. The aims of this study are to explore the effects of AGEs on the proliferation and function of ASCs and to assess whether DNA methylation plays a key role in these processes during diabetic osteoporosis.

## 2 MATERIALS AND METHODS

### 2.1 Materials

Advanced glycation end product (AGE)‐BSA was purchased from BIOVISION (San Francisco, USA). Osteogenic differentiation medium used for C57BL/6 mouse adipose‐derived stem cells (ASCs) was obtained from CYAGEN (Shanghai, China). The 5‐bromo‐4‐chloro‐3‐indolyl phosphate (BCIP)/nitro blue tetrazolium (NBT) alkaline phosphatase (ALP) colour development kit was purchased from BEYOTIME (Jiangsu, China). Primary antibodies were purchased from Abcam (Cambridge, UK), and secondary antibodies were provided by Thermo Fisher Scientific (MA, USA).

### 2.2 Diabetic osteoporosis animal model

Four‐week‐old female C57BL/6 mice were purchased from the Sichuan University Animal Experimental Center. All C57BL/6 mouse procedures were reviewed and approved by the Southwest Medical University Ethical Committee (20180391222). The animal experiments were conducted in accordance with the guidelines of the Care and Use of Laboratory Animals (Ministry of Science and Technology of China, 2006). Twenty 6‐week‐old C57BL/6 male mice were fed on a high fat and high sugar diet for one month. Their body weights were 25‐28 g, and they were divided into two groups randomly. The weight and blood sugar of the two groups were recorded every 3 days. After being fasted for 12 hours, the mice in the test group were intraperitoneally injected with streptozotocin (STZ, 140 mg/Kg, 5‐7 times/day, 1‐3 times in general), and the negative control group was injected with the same volume of sodium citrate buffer (1 mL/Kg). The fasting blood glucose of mice was monitored every 3 days after injection. When the fasting blood glucose of the test group remained higher than 11.1 mmol/L, the mice were considered diabetic.

### 2.3 ELISA

Furthermore, we randomly selected three diabetic mice and negative control mice for ELISA. The expression levels of AGEs in adipose tissue and bone tissue of the two groups were detected by sandwich enzyme‐linked immunosorbent assay (ELISA). The samples were added to the enzyme‐labelled well, which were pre‐labelled with anti‐AGE monoclonal antibodies, incubated, washed and labelled with biotinylated anti‐AGE antibody. Streptomycin‐HRP was added to washed wells and developed in the dark. The reaction was stopped with acid, and the optical density was measured at 450 nm using a spectrophotometer.

### 2.4 Micro‐CT analysis

Femurs were dissected from diabetic and control mice at week 24. Samples were fixed with 4% paraformaldehyde and analysed using a SCANCO Medical CT‐40 (SCANCO Medical, Switzerland). The whole femur was scanned completely. The main observation area was about 1 cm from the distal femur to the epiphysis.

### 2.5 Immunohistochemistry

The femur and tibia were dissected from 18‐week‐old mice. Immunohistochemical analyses were carried out according to the manufacturer's recommended protocol. The samples were incubated with mouse polyclonal anti‐5‐methylcytosine (5‐mC) (1:400, Abcam, UK). The sections were stained by 3,3′‐diaminobenzidine DAB kit (ZLI‐9018, ZSGB‐BIO, China). Stained sections were visualized under a light microscope (Olympus BX43F; JEOL, Tokyo, Japan).

### 2.6 Isolation and culture of ASCs, and the identification and characterization procedure of isolated ASCs

Adipose‐derived stem cells were collected from the subcutaneous fat of the inguinal sites. The adipose tissue was finely cut into smaller samples and treated with 0.075% type I collagenase (Sigma‐Aldrich, St. Louis, MO, USA) for 30 minutes. The mixture was centrifuged at 200 × *g* for 5 minutes, and the supernatant was subsequently removed. Culture medium consisting of α‐MEM (HYCLONE, Pittsburgh, USA), 10% foetal bovine serum (FBS) and 100 U/mL penicillin‐streptomycin (HYCLONE, Pittsburgh, USA) was added to the cell pellet of the ASCs. Finally, the ASCs were cultured in flasks under a standard humidified atmosphere of 5% CO_2_ at 37°C. Cells were sub‐cultured (1:3) when reaching 70‐80% confluence, and we could obtain purified ASCs at passage 3.

Third‐passage ASCs were resuspended as individual cell suspensions in phosphate‐buffered saline (PBS) (plus 10% FBS). One set of the test tube cells was stained with fluorophore‐conjugated antibodies to CD29, CD31 or CD45, and other tubes without fluorophore antibodies were usedas controls. We used FITC anti‐mouse CD29, CD45 and PE anti‐mouse CD31 antibodies (Biolegend, San Diego, CA, USA). Tubes with CD29, CD45 and CD31 were then cultured in the dark environment at room temperature for 30 minutes and then washed with PBS (plus 10% FBS). Finally, cells were mixedwith PBS (plus 10% FBS) in all tubes separately and detected with a fluorescence‐activated cell sorter (FACS Calibur; BD Biosciences, San‐Jose, CA, USA). The data were analysed using WinMDI2.8 software (The Scripps Institute, West Lafayette, IN, USA).^12^, ^26^, ^27^, ^28^

Furthermore, the osteogenic, adipogenic and chondrogenic differentiation ability of ASCs was tested.^19^, ^26^ ASCs were passaged to the third generation to obtain relatively pure ASCs cultures. For osteogenic induction, ASCs were seeded into the wells of 6‐well plates and cultured in osteogenic medium (Cyagen; USA). After 21 days, cells were washed twice with PBS, fixed in 4% paraformaldehyde for 1 hour and then incubated with Alizarin red‐S for 30 minutes. Cells were observed and imaged through an inverted phase contrast microscope (Nikon; Japan). For adipogenic induction, ASCs were seeded into the wells of 6‐well plates and cultured in adipogenic medium (Cyagen). After 14 days, cells were washed twice with PBS, fixed in 4% paraformaldehyde for 1 hours and then incubated with 0.3% Oil Red O for 30 minutes. Cells were observed and imaged through an inverted phase contrast microscope. For cartilage induction, ASCs were centrifuged in centrifuge tubes without resuspending and cultured in cartilage medium (Cyagen). After 21 days, cell aggregates were washed twice with PBS, fixed in 4% paraformaldehyde for 1 hour and imaged through a stereo fluorescence microscope (Carl Zeiss Microscopy; Germany). The cell balls were embedded in paraffin and stained by Alcian blue. Sections of the cell balls were observed and imaged through an optical microscope.

### 2.7 Cell proliferation assay

Cell counting kit‐8 (CCK‐8, DOJINDO, Shanghai, China) and real‐time cell analysis (RTCA) (x‐CELLIGENCE system, Roche Diagnostics GmbH, Basel, Switzerland) were used to detect the growth of ASCs. The second‐passage ASCs (5 × 10^4^ cells per 1 mL) were seeded into 96‐ and 16‐well plates. ASCs were cultured for 24 hours in 5% CO_2_ at 37°C; then, ASCs were treated with different concentrations of AGEs (20, 40, 80 and 160 µg/mL). Cell proliferation was measured by CCK‐8 for 24, 48 and 96 hours and monitored by RTCA for 24 hours. Then, the optical densities of CCK‐8 were measured at 450 nm by a microplate reader (Spectra Thermo, Switzerland), and data were analysed using the provided RTCA software.

### 2.8 Osteogenic induction

When first‐passage ASCs reached a fusion of 90%, second‐passage ASCs were seeded into 6‐well plates at a density of 2 × 10^4^ cells/cm. After culturing for 24 hours, α‐MEM medium was replaced by osteogenic differentiation medium, which included C57BL/6 mouse adipose‐derived stem cell osteogenic differentiation basal medium, 10% C57BL/6 mouse adipose‐derived stem cell osteogenic differentiation foetal bovine serum, 1% penicillin‐streptomycin, ascorbate (5 μmol/L), β‐glycerophosphate (10 mmol/L), dexamethasone (100 nmol/L) and 1% glutamine. Subsequently, cells were treated with AGEs at different concentrations during the osteogenic differentiation period.

### 2.9 Alkaline phosphatase and Alizarin red staining

Cells were treated as previously described. After osteogenic induction for 7 days, we used a BCIP/NBT alkaline phosphatase colour development kit to detect alkaline phosphatase. The steps of alkaline phosphatase staining were as follows: cells were rinsed with phosphate‐buffered saline (PBS) when the osteogenic differentiation medium was discarded. Then, cells were fixed with 4% paraformaldehyde at 4°C for 20 minutes and washed three times with PBS. BCIP/NBT alkaline phosphatase colour buffer solution was incubated at 37°C for 1 hour. The cells were rinsed with PBS three times. Finally, cells were observed and photographed under fluorescent microscope.

Calcium deposition is a late marker of ASCs osteogenic differentiation.^29^, ^30^ In brief, cells were washed three times in PBS after 14 days of osteogenic induction. Then, cells were fixed with 4% paraformaldehyde at 4°C for 20 minutes and washed three times with PBS. Alizarin Red dye (C57BL/6 mouse Adipose‐derived Stem Cell Osteogenic Differentiation Medium Kit, CYAGEN, Shanghai, China) was incubated for 15 minutes at 37°C, and cells were rinsed with PBS. Finally, cells were observed and photographed under a fluorescent microscope.

### 2.10 Immunofluorescence and confocal laser scanning microscopy

Cells were treated as previously described. After osteogenic induction for 7 days, the medium was discarded and cells were rinsed three times with PBS. Cells were fixed with 4% paraformaldehyde solution for 20 minutes, permeabilized with 0.5% TritonX‐100 for 20 minutes and blocked with 5% sheep serum at 37°C for 1 hour. Cells were washed with PBS three times after each step. Then, Runx2 (1:100; Abcam, Cambridge, UK), 5‐Methylcytosine (5‐mC; 1:100; Active Motif, Carlsbad, North America) rabbit monoclonal antibodies were incubated with the cell samples overnight at 4°C, and an appropriate fluorescence‐conjugated secondary labelled anti‐rabbit antibody (1:500; BEYOTIME, Shanghai, China) was subsequently added to bind with the primary antibody. DAPI was used to stain the nucleus, and phalloidine was applied to stain the cytoskeleton. Finally, images were captured using a confocal laser microscope (TCS SP8; Leica, WETZLAR, Germany).

### 2.11 Extraction of RNA and semi‐quantitative polymerase chain reaction

Expression levels of the following genes were detected by semi‐quantitative PCR after ASCs were treated as described before osteopontin (OPN), runt‐related transcription factor 2 (Runx2), alkaline phosphatase (ALP), glycogen syntheses kinase (GSK), lymphatic enhancement factor‐1 (Lef‐1) and β‐catenin. The primer sequences used to detect the relevant genes are displayed in (Table 1). RNeasy plus mini kit (Qiagen, CA, USA) was used for the extraction and purification of total RNA. The final volume of cDNA was 20 μL, and the preparation of cDNA was performed in accordance with the instructions in the cDNA synthesis kit (MBI, Glen Burnie, MD, USA). A real‐time PCR system (Bio‐Rad, Hercules, CA, USA) combined with a PCR kit (MBI, Glen Burnie, MD, USA) was used for the amplification of the relevant genes. The DNA samples were subjected to electrophoresis on a 2% agarose gel, and the bands were visualized by staining with Gold View (Heart, Shanghai, China). Glyceraldehyde‐3‐phosphate dehydrogenase (GAPDH) and beta‐actin (β‐actin) were used as the internal control and normalized standard, respectively. Image‐Pro plus 6.0 (Media Cybernetics, Rockville, MD, USA) was used to detect the optical density of each band.

**Table 1:** Sequences of the PCR primers for amplification of expressed genes

Genes	Sequence(5′→3′)
*Gapdh*	F: GGTGAAGGTCGGTGTGAACG R: CTCGCTCCTGGAAGATGGTG
*Alp*	F: GAGGCATACGCCATCACATG R: CCGATGGCACACCTGCTT
*Opn*	F: GGATTCTGTGGACTCGGATG R: CGACTGTAGGGACGATTGGA
*Ocn*	F: AGCAGCTTGGCCCAGACCTA R: TGAGGCTCCAAGGTAGCGCC
*Runx2*	F: CCGAACTGGTCCGCACCGAC R: CTTGAAGGCCACGGGCAGGG
*Dnmt1*	F: ATCCTGTGAAAGAGAACCCTGT R: CCGATGCGATAGGGCTCTG
*Dnmt3a*	F: GAGGGAACTGAGACCCCAC R: CTGGAAGGTGAGTCTTGGCA
*Dnmt3b*	F: AGCGGGTATGAGGAGTGCAT R: GGGAGCATCCTTCGTGTCTG

### 2.12 Western blot assay

The treated ASCs were rinsed with cold PBS three times, and total proteins were harvested with a cell protein extraction reagent (KEYGEN Biotech, Nanjing, China). The collected proteins were then mixed with loading buffer at a ratio of 4:1 (V/V) and boiled for 5 minutes. The proteins were separated on a 10% SDS‐PAGE, and proteins were subsequently transferred onto a polyvinylidene fluoride membrane. After blocking with 5% skim milk for 1 hour at 37°C, the membranes were incubated with primary rabbit monoclonal antibodies specific to the following targets: OPN, Runx2, Lef‐1, GSK, cyclin D and β‐catenin (Abcam, Cambridge, UK). Then, each band was incubated with a secondary labelled anti‐rabbit antibody (BEYOTIME, Shanghai, China), and the results were visualized using an ECL chemiluminescence detection system (Bio‐Rad, Hercules, CA, USA).

### 2.13 Statistical analysis

All experiments were performed in triplicate and reproduced at least three times. Student's *t* test or one‐way ANOVA followed by Duncan's multiple range tests was used for statistical analysis. Statistical analysis was completed using SPSS 18.0.

## 3 RESULTS

### 3.1 Diabetic osteoporosis mice

**Figure 1:**
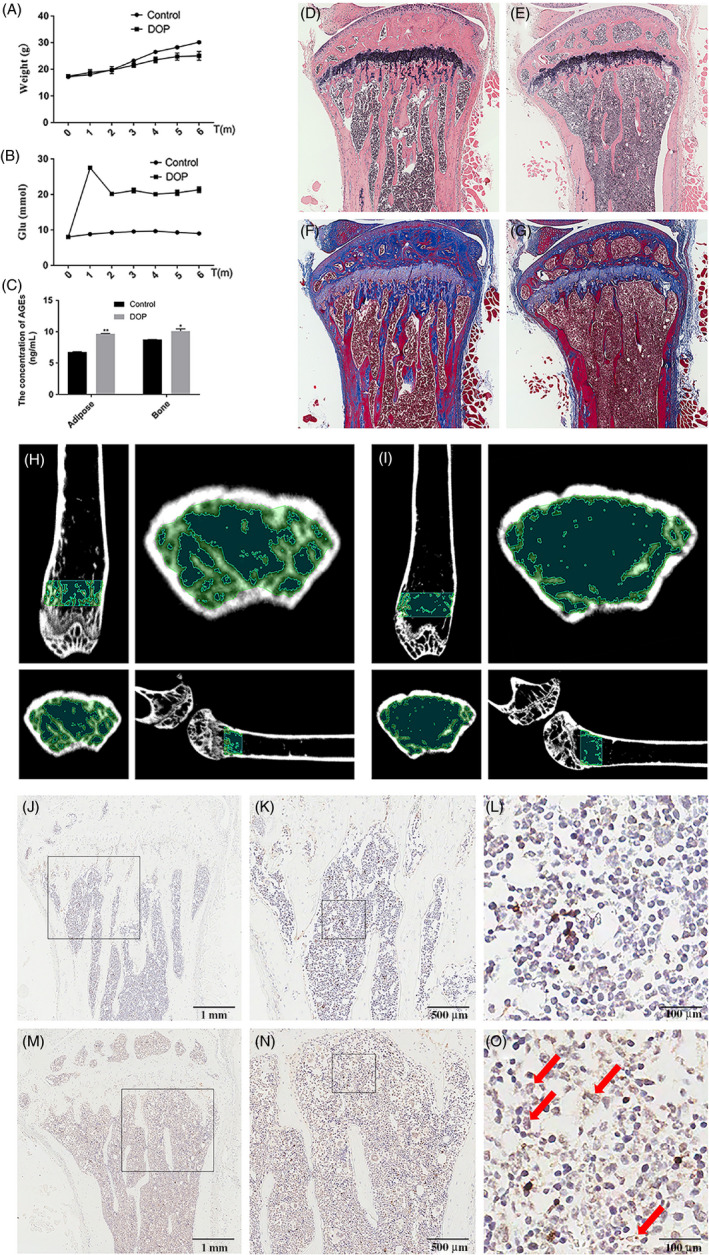
Diabetic osteoporosis mice with increased concentration of AGEs and DNA methylation levels in the tibia exhibit reduced alveolar bone mass and density. A, B, The levels of blood glucose and weight in mice with high fat and sugar (the control group) and diabetic osteoporosis mice for 6 months. C, The content of AGEs in adipose and bone tissue was detected by ELISA. Data are presented as mean ± SD (n = 4). Student's *t* test is used for statistical analysis. Statistical analysis: *P < .05, **P < .01. D‐G, Reduced alveolar bone mass and density in HE/Masson images of 24‐week‐old diabetic osteoporosis mice (right) compared with control mice. H, I, Reduced alveolar bone mass and density in micro‐CT images of 24‐week‐old diabetic osteoporosis mice (right) compared with control mice. J‐O, Immunohistochemical analysis of 5‐mC expression in 24‐week‐old diabetic osteoporosis mice bone tissues (M–O) compared with control group (J–L). Red arrows show positive staining of 5‐mC expression in diabetic osteoporosis mice bone tissues

We established a model of diabetic osteoporosis in C57BL/6 mice. There were significant differences between the diabetic osteoporosis group and healthy controls. In the diabetic osteoporosis group, blood glucose levels were maintained above 20 mmol/mL, but the body weight of the mice was slightly lower than the control group (Figure 1A,B). The ELISA results depicted an increase in AGEs in adipose and bone tissue in the diabetic osteoporosis group compared with controls (Figure 1C). Moreover, haematoxylin and eosin and Masson staining data demonstrated that the number of bone trabeculae decreased, the morphology of bone trabeculae became smaller and discontinuous, and the trabecular space widened in the diabetic osteoporosis group (Figure 1D‐G). These results were further confirmed by Micro‐CT analysis (Figure 1H,I). Immunohistochemistry staining showed that 5‐mC, the main product of DNA methylation used to indicate the extent of methylation, was dramatically increased in the diabetic osteoporosis group (Figure 1M‐O) compared with the control group (Figure 1J‐L). Taken together, our results demonstrated that bone mass and density were decreased with increased concentration of AGEs and DNA methylation in diabetic osteoporosis mice.

### 3.2 Cell proliferation

**Figure 2:**
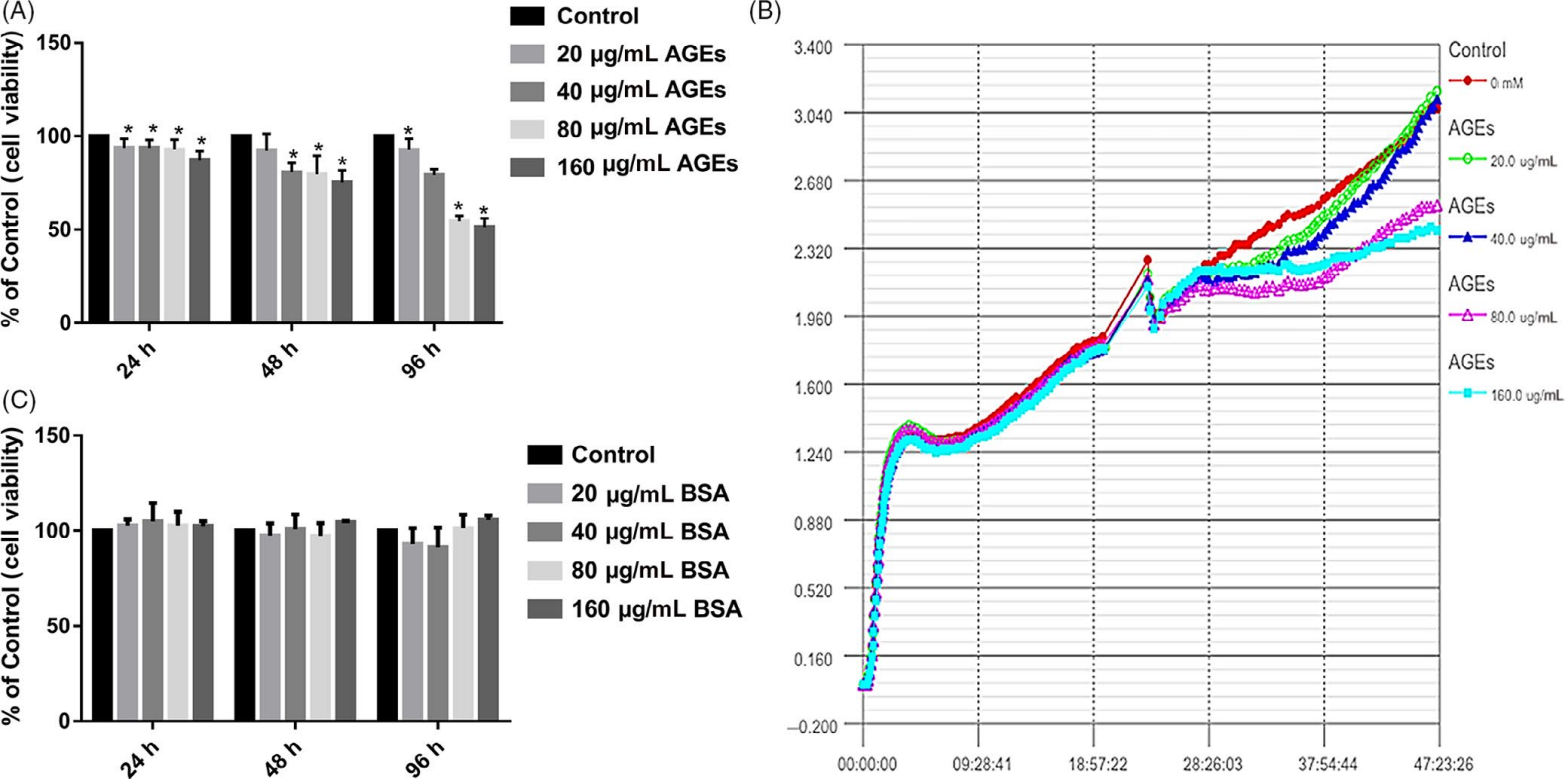
Effect of AGEs on cell proliferation of ASCs. A, Cells were treated with AGEs at various concentrations for 24, 48 or 96 h, and cell viability was estimated using CCK‐8. Values represent mean SE of at least three independent experiments. *P < .05 versus control. B, Cell proliferation was detected by real‐time cell analysis system (RTCA). (C) Cells were treated with BSA at various concentrations for 24, 48 or 96 h, and cell viability was estimated using CCK‐8. Values represent mean SE of at least three independent experiments

CCK‐8 results showed that AGEs inhibit the proliferation of ASCs. The ASCs were still highly proliferative (80% activity) following treatment with low concentrations of AGEs (20 or 40 μg/mL) for 24, 48 and 96 hours while treatment with high concentrations of AGEs (80 or 160 μg/mL) for 24, 48 and 96 hour significantly inhibited cell proliferation, particularly at 160 μg/mL AGEs for 96 hours (50% activity) (Figure 2A). The control BSA at various concentrations did not have a significant effect on cell viability (Figure 2C). RTCA (Figure 2B) was consistent with the results of the CCK‐8 assays, demonstrating that AGEs inhibited the proliferation of ASCs in a dose‐dependent manner.

### 3.3 AGEs inhibit ASCs osteogenic differentiation potential

**Figure 3:**
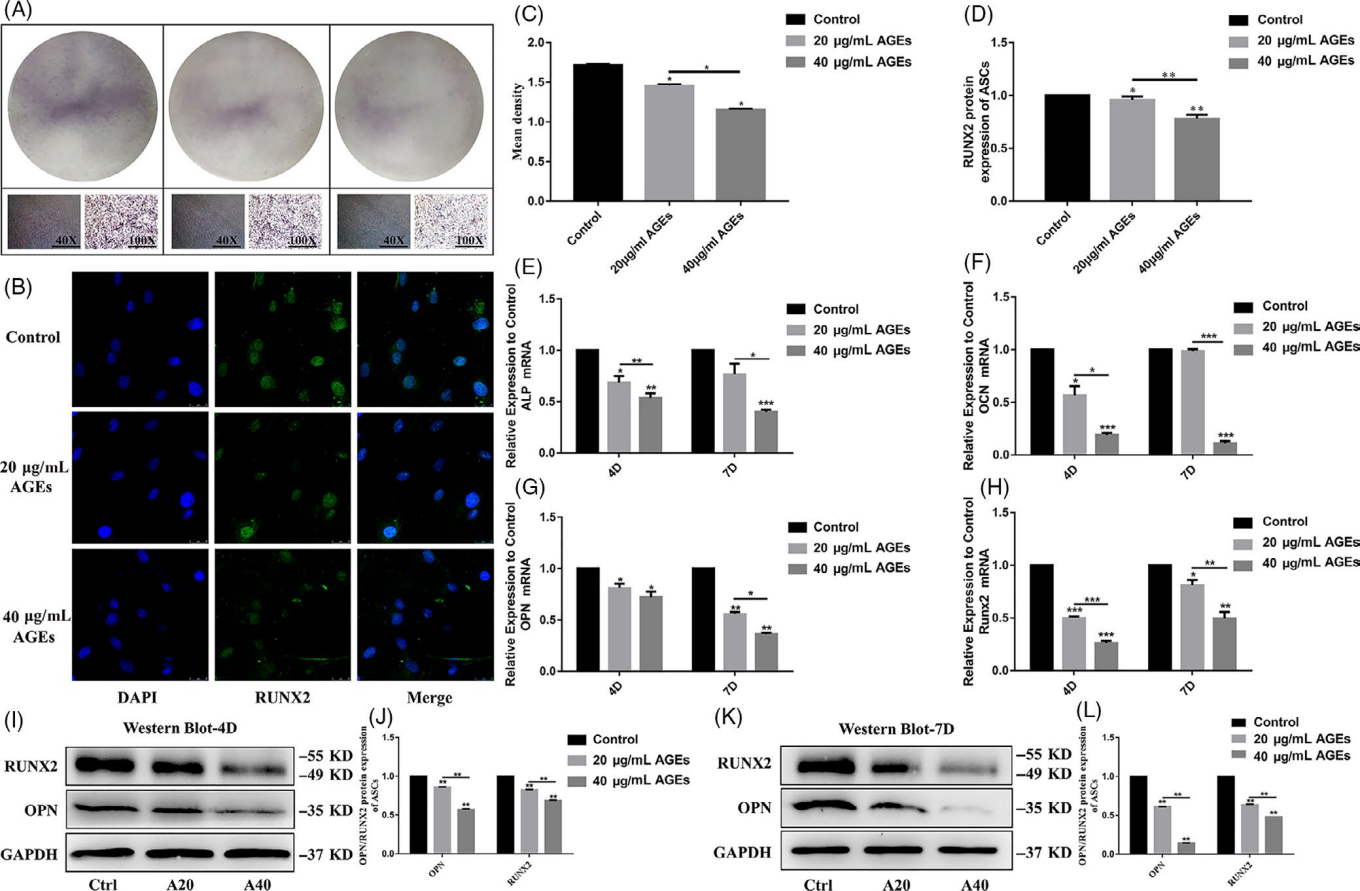
Effect of AGEs on osteogenic differentiation of ASCs. A, Osteogenic differentiation was detected by ALP staining at day 7. C, Semi‐quantitative analysis of ALP. Data are presented as mean ± SD (n = 4). Statistical analysis: * *P* < .05. B, Interaction of ASCs with 20 and 40 μg/mL AGEs for 4D (RUNX2: green, nucleus: blue). Scale bars are 25 µm. D, Semi‐quantitative analysis of fluorescence of RUNX2. Data are presented as mean ± SD (n = 4). Statistical analysis: *P < .05, **P < .01. E‐H, Quantitative real‐time PCR analysis of the osteogenic‐specific gene expression (GAPDH was used as the internal control). Data are presented as mean ± SD (n = 4). Student's *t* test is used for statistical analysis. Statistical analysis: *P < .05, **P < .01, ***P < .001. I, K, Western blot analysis of OPN and RUNX2 expression levels (GAPDH was used as internal control) after we treated ASCs with 20 and 40 µg/mL AGEs in osteogenic induction culture for 4 and 7 days. J, L, Quantification of OPN and RUNX2 expression levels, GAPDH levels set as internal normalized control. Student's *t* test is used for statistical analysis. Data are presented as mean ± SD (n = 4). Statistical analysis: *P < .05, **P < .01, ***P < .001

Alkaline phosphatase activity, an early osteogenic differentiation marker, was measured using a BCIP/NBT alkaline phosphatase colour development kit at day 7 during osteogenic differentiation. Compared with the non‐treated group, ALP activity was significantly decreased with less NBT‐formation in ASCs treated with 20 and 40 μg/mL AGEs during osteogenic induction culture for 7 days (Figure 3A,C). Furthermore, the activity of ALP decreased with the increase of AGEs concentration.

The gene expression of *Alp, Opn, Ocn* and *Runx2* was assessed after treatment with AGEs, and the mRNA levels of *Alp, Opn, Ocn* and *Runx2* in the AGEs‐treated group were significantly lower than the control group. Additionally, as the concentration of AGEs increased, the expression of osteogenic‐specific genes decreased. The mRNA levels of *Alp, Opn, Ocn* and *Runx2* in the 40 μg/mL AGEs‐treated group were significantly lower than that in the 20 μg/mL AGEs‐treated group (Figure 3E‐H).

After treatment with 20 or 40 μg/mL AGEs for 4 days, immunofluorescence staining showed that the protein expression level of RUNX2 in the ASCs was lower than in the control group. Furthermore, the expression of RUNX2 in the 40 μg/mL AGEs group was significantly lower compared with the 20 μg/mL AGEs group (Figure 3B,D). The expression of OPN and RUNX2 protein was significantly decreased after treatment with AGEs as detected by Western blot analysis (Figure 3I,K). Meanwhile, statistical analysis further confirmed the results as in ASCs treated with 20 or 40 μg/mL AGEs, OPN was 0.86‐fold and 0.57‐fold less and Runx2 was 0.82‐fold and 0.68‐fold less than the control group after 4 days, respectively. After 7 days, OPN was 0.61‐fold and 0.14‐fold less and RNUX2 was 0.63‐fold and 0.47‐fold less than controls (Figure 3J,L). Figure 3 demonstrates that AGEs inhibit ASCs osteogenic differentiation potential in a dose‐dependent manner.

### 3.4 AGEs increase DNA methylation in ASCs

**Figure 4:**
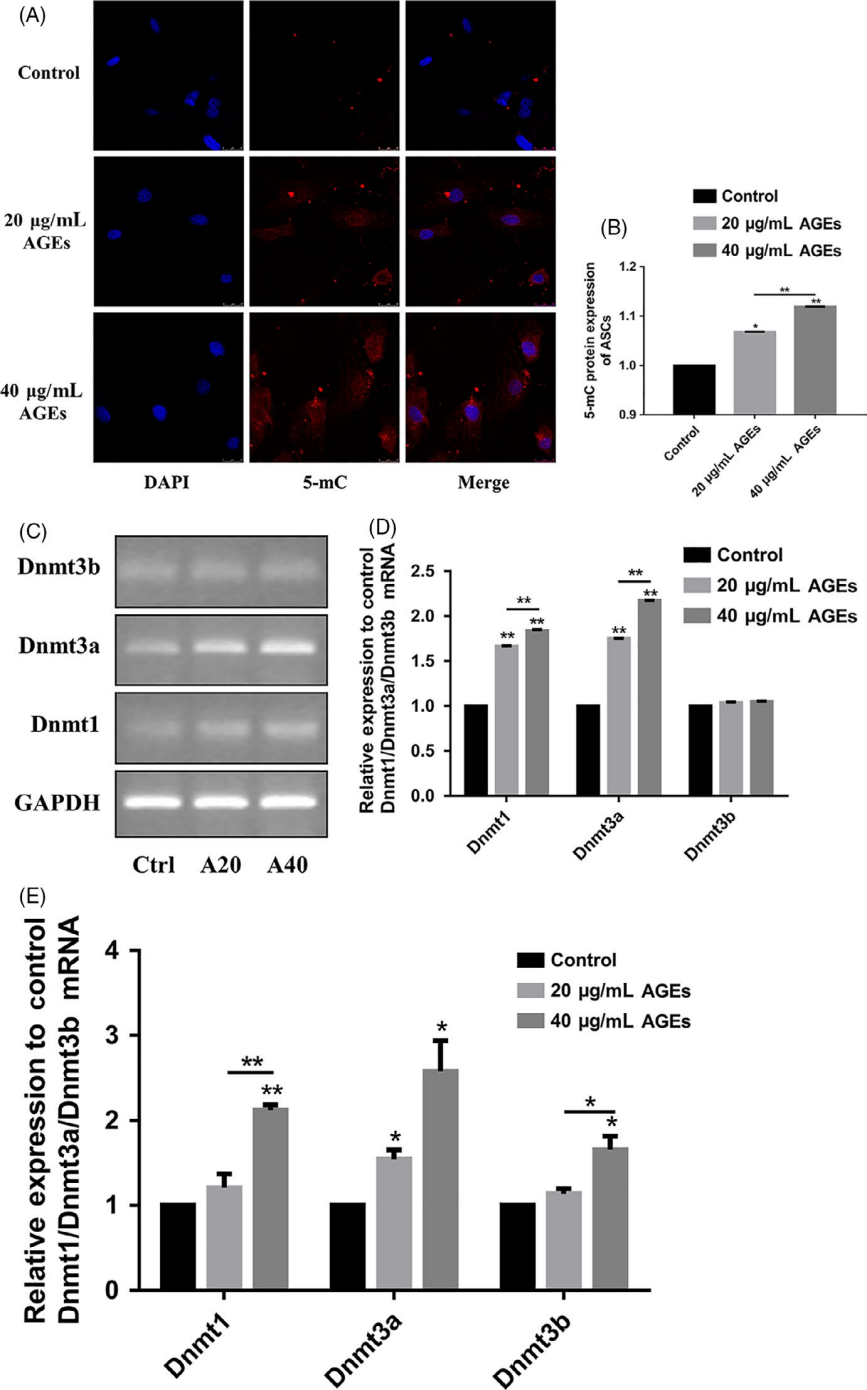
Effect of AGEs on DNA methylation of ASCs. A, Interaction of ASCs with 20 and 40 μg/mL AGEs for 4D (5‐mC: red, nucleus: blue). Scale bars are 25 µm. B, Semi‐quantitative analysis of fluorescence of 5‐mC. Data are presented as mean ± SD (n = 4). Student's *t* test is used for statistical analysis. Statistical analysis: *P < .05, **P < .01. C, D, Semi‐quantitative PCR analysis of the DNA methylation‐specific gene expression (GAPDH was used as the internal control). Data are presented as mean ± SD (n = 4). Student's *t* test is used for statistical analysis. Statistical analysis: **P < .01. E, Quantitative real‐time PCR analysis of the DNA methylation‐specific gene expression (GAPDH was used as the internal control). Data are presented as mean ± SD (n = 4). Student's *t* test is used for statistical analysis. Statistical analysis: *P < .05, **P < .01

The main product of DNA methylation in ASCs, 5‐mC, was detected using immunofluorescence after treatment with 20 and 40 μg/mL AGEs for 4 days. We found that with increasing AGEs concentration, the expression of 5‐mC increased (Figure 4A). The statistical analysis of immunofluorescence images confirmed that the expression of 5‐mC in ASCs treated with 40 μg/mL AGEs was expressed as much as 1.12‐fold compared with the control group (Figure 4B).

The mRNA expression of *Dnmt1, Dnmt3a* and *Dnmt3b* was analysed using semi‐quantitative PCR and quantitative RT‐PCR. As shown in Figure 3C, treatment with 20 or 40 μg/mL AGEs resulted in a significant increase in expression of DNMTs (Figure 4C). The statistical analysis further confirmed that *Dnmt1* increased by 1.84‐fold, *Dnmt3a* increased by 2.18‐fold and *Dnmt3b* increased by 1.05‐fold (Figure 4D). Furthermore, the results of the quantitative RT‐PCR analysis (Figure 4E) were consistent with the semi‐quantitative PCR analysis, demonstrating that AGEs increase the DNA methylation level in ASCs, particularly *Dnmt1* (increased 2.12‐fold) and *Dnmt3a* (increased 2.58‐fold). Taken together, these results demonstrated that AGEs increased the DNA methylation level in ASCs.

### 3.5 DNMT inhibitor 5‐aza‐dC rescues the osteogenic differentiation capacity of ASCs treated with AGEs

**Figure 5:**
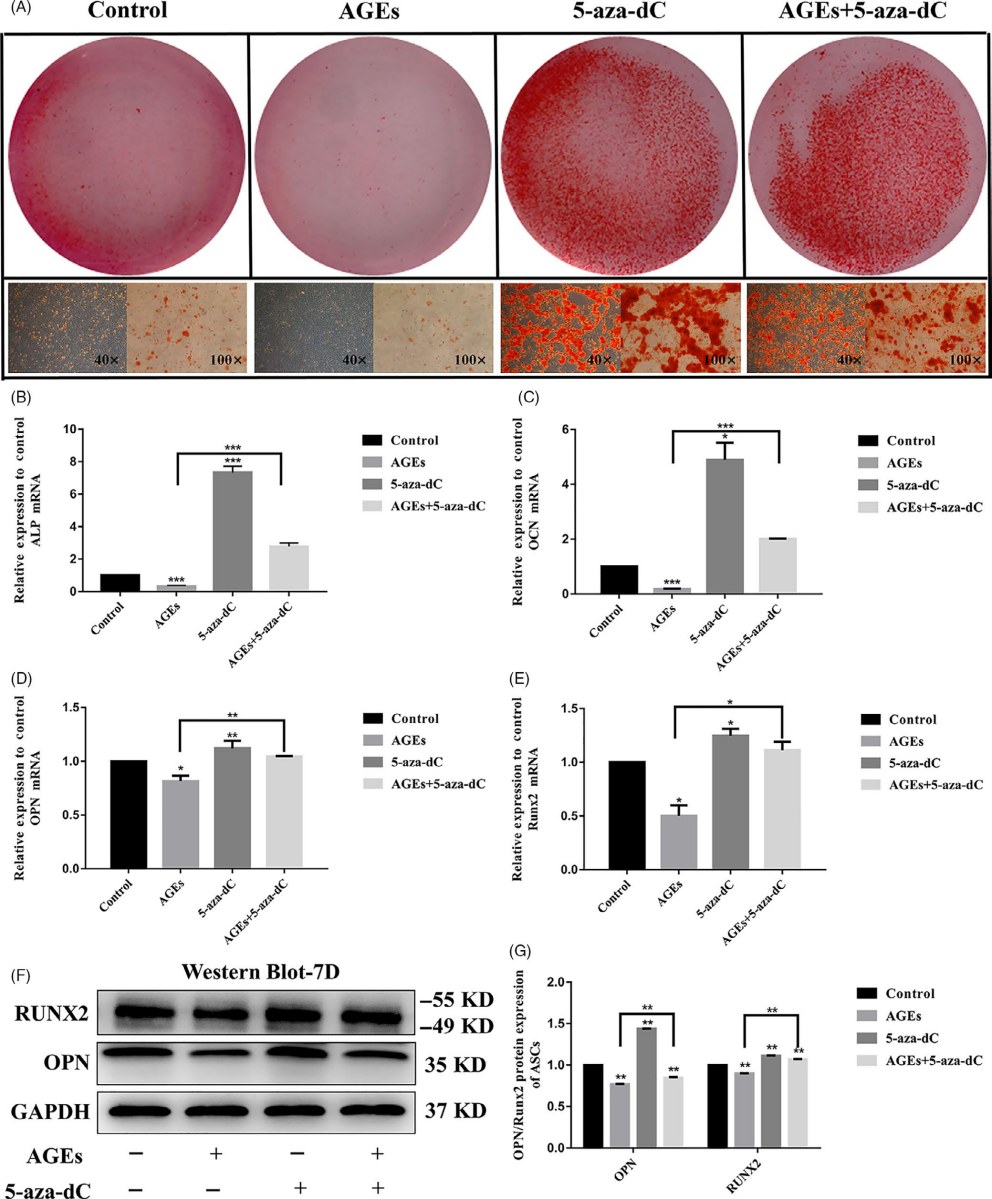
5‐aza‐dC rescues the osteogenic differentiation capacity of ASCs treated with μg/mL AGEs. A, Osteogenic differentiation was detected by Alizarin Red staining at day 14. B‐E, Quantitative real‐time PCR analysis of the osteogenic‐specific gene expression (GAPDH was used as the internal control). Data are presented as mean ± SD (n = 4). Student's *t* test is used for statistical analysis. Statistical analysis: *P < .05, **P < .01, ***P < .001. F, G, Western blot analysis of OPN and RUNX2 expression levels (GAPDH was used as internal control) and quantification of OPN and RUNX2 expression levels. GAPDH levels set as internal normalized control. Student's *t* test is used for statistical analysis. Data are presented as mean ± SD (n = 4). Statistical analysis: **P < .01

Subsequently, we explored the association between DNA methylation and the osteogenic differentiation potential of ASCs. The DNA methylation inhibitor 5‐aza‐dC inhibited *Dnmt1* during cell replication and prevented the methylation of new chains, leading to demethylation.^19^ Alizarin red staining showed that treatment of ASCs with 40 μg/mL AGEs resulted in fewer mineralized nodules than in the control group (Figure 5A), consistent with previous results. In contrast, ASCs incubated with 1 μm of the DNMT inhibitor, 5‐aza‐dC, produced more mineralized nodules than the control group (Figure 5A). We also found that there were more mineralized nodules after treatment with 40 μg/mL AGEs and 1 µm 5‐aza‐dC than in the AGEs group (Figure 5A). 5‐aza‐dC rescued the osteogenic differentiation capacity of ASCs treatment with AGEs. To further assess the effect of the AGEs and 5‐aza‐dC treatment on osteogenic differentiation in ASCs, we detected the mRNA levels of *Alp, Opn, Ocn* and *Runx2*. The expression of these genes after treatment with AGEs was significantly down‐regulated (*Alp* was decreased by 0.33‐fold, *Opn* was decreased by 0.81‐fold, and *Ocn* was decreased by 0.18‐fold), while treatment with 5‐aza‐dC led to an increase in the expression of these osteogenic‐related genes compared with controls (Figure 5B‐E). *Alp* was increased by 7.34‐fold, *Opn* increased by 1.12‐fold, *Ocn* increased by 3.8‐fold, and *Runx2* increased by 1.25‐fold. The expression levels of the osteogenic‐related genes in the AGEs and 5‐aza‐dC‐treated group were higher than in ASCs treated with AGEs alone (40 μg/mL; *Alp* was increased by 2.78‐fold, *Opn* was increased by 1.04‐fold, *Ocn* was increased by 2‐fold, and *Runx2* was increased by 1.11‐fold: Figure 5B‐E). 5‐aza‐dC increased the low expression levels of these osteogenic‐related genes after treatment with AGEs. We also assessed the expression of protein levels by Western blot. Western blot analysis (Figure 5F, G) was consistent with results from quantitative RT‐PCR, demonstrating that 5‐aza‐dC rescued the osteogenic differentiation capacity of ASCs treatment with AGEs.

### 3.6 The canonical Wnt signalling pathway is involved in 5‐aza‐dC‐induced osteogenic differentiation in ASCs treated with AGEs

**Figure 6:**
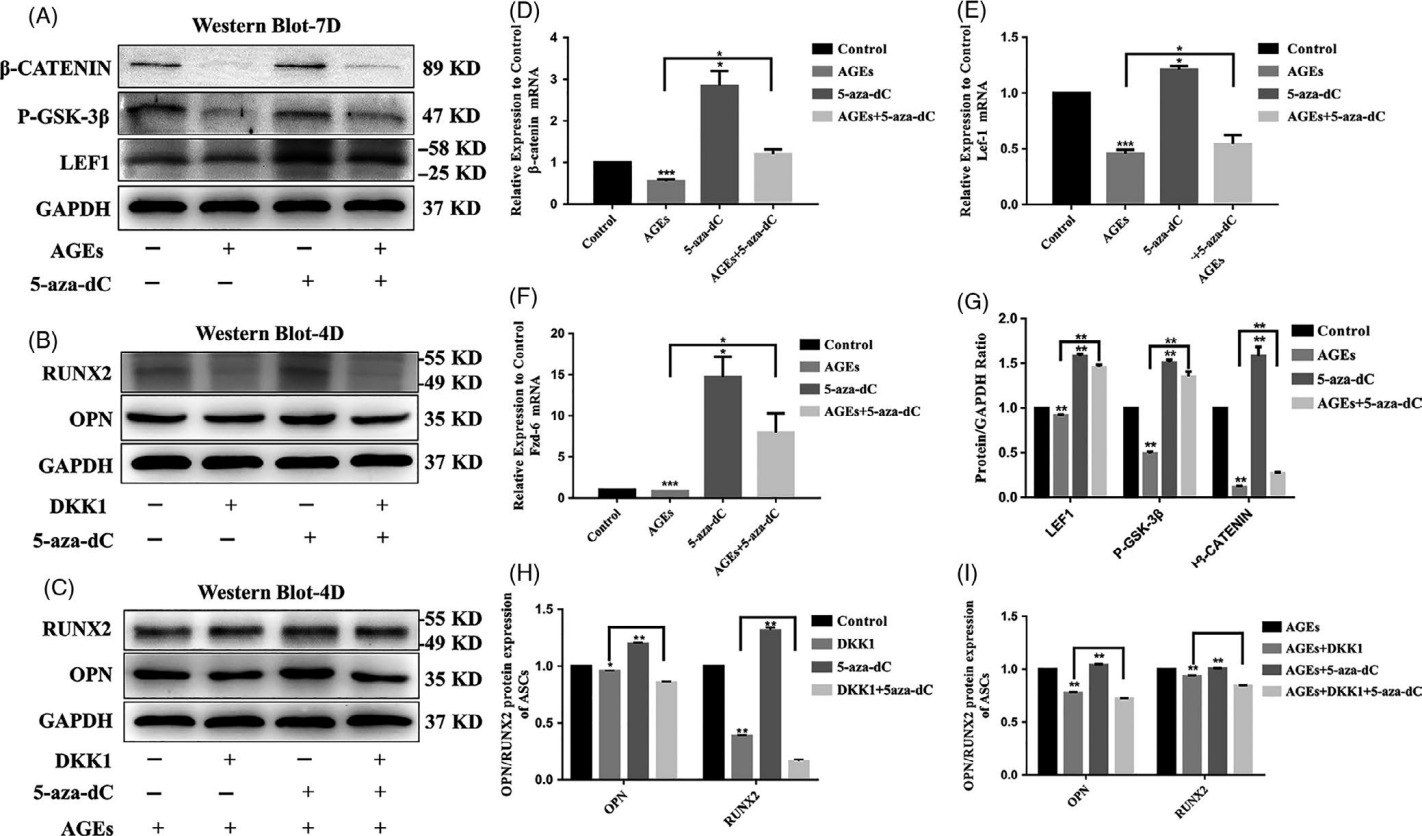
The canonical Wnt signalling pathway is involved in 5‐aza‐dC‐induced osteogenic differentiation of ASCs treated with AGEs. A, G, Western blot analysis of LEF1, P‐GSK‐3β and β‐CATENIN expression levels (GAPDH was used as internal control) and quantification of LEF1, P‐GSK‐3β and β‐CATENIN expression levels. GAPDH levels set as internal normalized control. Student's *t* test is used for statistical analysis. Data are presented as mean ± SD (n = 4). Statistical analysis: *P < .05, ***P < .001. D‐F, Quantitative real‐time PCR analysis of the canonical Wnt signalling pathway‐specific gene expression (GAPDH was used as the internal control). Data are presented as mean ± SD (n = 4). Student's *t* test is used for statistical analysis. Statistical analysis: **P < .01. B, H, Canonical Wnt signalling pathway inhibitor DKK1 attenuates the osteogenesis of ASCs with 5‐aza‐dC. Western blot analysis of OPN and RUNX2 expression levels (GAPDH was used as internal control). Data are presented as mean ± SD (n = 4). Student's *t* test is used for statistical analysis. Statistical analysis: **P < .01. C, I, Canonical Wnt signalling pathway inhibitor DKK1 attenuates the osteogenesis of ASCs with 40 μg/mL AGEs and 5‐aza‐dC. Western blot analysis of OPN and RUNX2 expression levels (GAPDH was used as internal control). Data are presented as mean ± SD (n = 4). Student's *t* test is used for statistical analysis. Statistical analysis: ** *P* < .01

The canonical Wnt signalling pathway plays a crucial role in osteogenesis. Therefore, we investigated the effect on Wnt/β‐catenin signalling on the osteogenic differentiation of ASCs. We detected the mRNA and protein expression levels of Wnt signalling molecules to investigate the role of AGEs and 5‐aza‐dC in osteogenesis in ASCs. After ASCs were incubated with osteogenic medium for 4 days, they were treated with AGEs and 5‐aza‐dC, the gene expression of *β‐catenin, Lef1* and *Fzd6* was determined using quantitative RT‐PCR analysis. As shown in Figure 6, treatment of ASCs with AGEs led to the largest decrease in the mRNA expression levels of the canonical Wnt signalling molecules compared with the other groups; *β‐catenin* decreased by 0.55‐fold, *Lef1* decreased by 0.45‐fold, and *Fzd6* decreased by 0.86‐fold. (Figure 6D‐F). While ASCs exposed to 1 μm 5‐aza‐dC showed the highest expression of the canonical Wnt signalling molecules than other groups, *β‐catenin* increased by 2.84‐fold, *Lef1* increased by 1.21‐fold, and *Fzd6* increased by 14.7‐fold. (Figure 6D‐F). Furthermore, 5‐aza‐dC resulted in recovered expression of the canonical Wnt signalling molecules after treatment with AGEs. The expression levels of Wnt‐related genes in cells treated with both AGEs and 5‐aza‐dC were higher than cells treated with AGEs alone; *β‐catenin* increased by 1.2‐fold, *Lef1* increased by 0.54‐fold, and *Fzd6* increased by 7.97‐fold (Figure 6D‐F). Additionally, we assessed the protein expression by Western blot. The results were consistent with the quantitative RT‐PCR results showing a decrease in Wnt‐related proteins following treatment with AGEs and a recovery of this decrease following treatment with 5‐aza‐dC (Figure 6A,G).

To further verify the involvement of canonical Wnt signalling in the regulation of osteogenesis in ASCs, DKK1 was used to inhibit canonical Wnt signalling pathway activity. Western blot analysis showed that in the presence of DKK1, 5‐aza‐dC‐induced expression of osteogenic differentiation‐related proteins was decreased (Figure 6B,H). This was also observed in the presence of AGEs (Figure 6C,I). Collectively, these results indicated that 5‐aza‐dC promoted the osteogenic differentiation of ASCs via the canonical Wnt signalling pathway.

## 4 DISCUSSION

Diabetic osteoporosis is a systemic metabolic bone disease where diabetes mellitus is accompanied by osteopenia. The destruction of bone microstructure occurs, and patients are prone to fractures.^1^, ^2^, ^3^ In our diabetic osteoporosis model, the lower bone density and the abnormal trabecular morphology were showed in diabetic osteoporosis mice than that in control group. Autologous transplantation of ASCs is used to promote the repair of bone defects and bone regeneration, which is widely used in the treatment of fractures and bone defects associated with diabetic osteoporosis. However, in a high glucose environment, the osteogenic differentiation ability of ASCs was suppressed, which leads to a lack of bone formation and poorer repair of bone tissue. In this study, we investigated the molecular mechanisms underlying the osteogenic differentiation of ASCs in the diabetic microenvironment.

Recent studies have confirmed that AGEs are involved in the development and progression of diabetic osteoporosis.^1^, ^14^, ^15^, ^16^, ^17^, ^18^ The formation and deposition of AGEs accelerate during ageing, inflammation and particularly diabetes, and it is associated with the development and progression of diabetes‐associated osteoporosis. Sun et al^16^ reported that AGEs inhibited the proliferation and osteogenic differentiation of rat mesenchymal stem cells. Chuguransky et al^17^ reported that alendronate improves bone alterations in experimental diabetes by preventing antiosteogenic, antichondrogenic and proadipocytic effects of AGEs on bone marrow progenitor cells. Our study showed that the content of AGEs in adipose tissue and bone tissue increased and the bone formation decreased during diabetic osteoporosis in mice. Furthermore, the RTCA and CCK‐8 results showed that AGEs inhibited the proliferation, and the Western blot and PCR data showed that the expression of osteogenic genes was decreased in AGEs‐treated group. Interestingly, we also found that the down‐proliferative effect of AGEs on ASCs not only depends on time and concentration in isolation but on the synergistic effects of both factors.

It has been widely reported that DNA methylation levels are associated with bone diseases in the diabetic microenvironment.^18^, ^19^, ^20^, ^21^, ^22^, ^23^, ^24^ In a recent study, Zhang et al^19^ reported that FPS‐ZM1 treatment rescued the loss of osteogenic differentiation in ASCs by inhibiting AGE‐induced DNA hypermethylation. Liu et al^20^ also showed that the DNA methylation levels of human periodontal ligament stem cells (hPDLSCs) increased in the diabetic microenvironment and 5‐aza‐dC improves the osteogenic differentiation potential of hPDLSCs. Kalwa et al^31^ found that LncRNA‐HOTAIR regulated the osteogenic differentiation of bone mesenchymal stem cells (BMSCs) by regulating the DNA methylation levels of histones by combining PRC2 and LSD1 complexes. In our study, ASCs cultured in AGEs‐containing medium expressed high levels of 5‐mC and DNMTs (DNMT1, DNMT3a and DNMT3b) and had reduced osteogenic differentiation in vitro. After the treatment with the DNMT inhibitor (5‐aza‐dC), the osteogenic differentiation potential of ASCs was improved by promoting DNA demethylation.

Studies showed that the canonical Wnt signalling pathway was activated during DNA methylation‐regulated metabolism.^32^, ^33^, ^34^, ^35^, ^36^, ^37^ Chiba et al^38^ showed that decreased DNA methylation in the promoter region of the WNT5A and GDNF genes may promote the osteogenicity of mesenchymal stem cells from patients with ossified spinal ligaments. Liu et al^39^ reported that AGEs inhibited the osteogenic differentiation of PDLSCs, while the adverse effect of AGEs in PDLSCs could be reversed when DKK1 was used to inhibit Wnt signalling. We demonstrated that the gene expressions of β‐catenin, Lef1 and P‐GSK‐3β were suppressed in AGEs‐treated group, while they were increased in 5‐aza‐dC‐treated group. Furthermore, 5‐aza‐dC treatment reversed the expression of Wnt proteins in AGEs‐treated group. Interestingly, we further demonstrated that 5‐aza‐dC could up‐regulate the osteogenic differentiation capacity of ASCs via the canonical Wnt signalling pathway, but DKK‐1 inhibited this process. Our results demonstrated that AGEs inhibited the osteogenic differentiation of ASCs by modulating the Wnt signalling pathway via DNA methylation in vitro.

In summary, our study demonstrated that DNA methylation was up‐regulated in ASCs cultured in AGEs‐supplemented media while the canonical Wnt signalling pathway and osteogenic differentiation were inhibited. Moreover, the treatment of DNMT inhibitor 5‐aza‐dC reversed the osteogenic differentiation by modulating the canonical Wnt signalling pathway via DNA demethylation in AGEs‐treated ASCs. Our work contributes to our understanding of the impaired osteogenic differentiation capacity of ASCs during diabetes and provides a possible treatment for bone tissue regeneration with diabetic osteoporosis.

## CONFLICT OF INTERESTS

The authors declare that there is no conflict of interests regarding the publication of this paper.

## AUTHOR CONTRIBUTION

All authors contributed to research concept. Yong Li and Lang Wang established diabetic osteoporosis animal model and carried out in vivo experiments. Yong Li, Lang Wang and Maorui zhang performed isolation and culture of ASCs, and in vitro experiments. Kui Huang, Zhihao Yao and Pengcheng Rao collected the data. Yong Li executed the analysis of the data and drafted the manuscript. Xiaoxiao Cai designed experimental project, analysed data and revised manuscript. Jingang Xiao initiated study, designed experimental project, analysed data, revised manuscript and provided funding. All authors have seen and approved the manuscript.

## Data Availability

The data which support research results are available from the corresponding author upon reasonable request.
